# Mental Health Act 1983 Assessments: Audit of Compliance With GMC and Legal Expectations

**DOI:** 10.1192/bjo.2025.10546

**Published:** 2025-06-20

**Authors:** Leila Ali, William Melton, Navjot Ahluwalia

**Affiliations:** Rotherham, Doncaster and South Humber NHS Foundation Trust, Doncaster, United Kingdom

## Abstract

**Aims:** Serious concerns have been raised by a HM Coroner in England regarding poor medical recording and communication following Mental Health Act Assessments (MHAA). We aimed to undertake a ‘deep-dive’ audit of documentation and inter-professional communication to assess compliance with GMC Good Medical Practice (GMP) and Mental Health Act Code of Practice 2015 (MHACP) following a MHAA in the Doncaster locality of Rotherham Doncaster and South Humber NHS Foundation Trust (RDASH).

**Methods:** Audit standards were set using the GMC GMP guidance and the MHACP. GMC GMP states ‘You must make sure that formal records of your work (including patient records) are clear, accurate, contemporaneous and legible’ and makes clear each doctor is responsible for recording their own independent medical opinion for any significant clinical intervention.

All patients detained on section 136 in September 2024 in the Doncaster locality were selected. The electronic records system (SystmOne) was scrutinised for (1) length of time taken for the assessment to be documented on SystmOne by any of the assessing team, (2) the number of doctors documenting the assessment, (3) whether any doctor communicated with the GP.

An email was sent to each patient’s GP regarding any communication they received from a doctor relating to the MHAA in case communication was not included on the internal Trust system.

Data was also collected on the outcome of the assessment, number of doctors involved, their roles and whether they were employed by the Trust.

**Results:** Of the 23 MHAAs, 18 took place in the Doncaster S136 suite and 5 in A&E. Nineteen assessments did not result in detention, 3 resulted in an informal admission, and 1 in detention.

Documentation on SystmOne took over 1 day for 61% (14/23) of MHAAs, all completed by an Allied Mental Health Professional (AMHP). In 26% (6/23) documentation occurred within 4 hours, all by an assessing doctor. In 4% (1/23) it was completed within 12 hours by an AMHP.

A total of 9% (2/23) had no AMHP report or documentation. In 70% (16/23) neither doctor documented, while in 30% (7/23) one doctor documented.

In only 22% (5/23) of MHAAs a letter was sent to the GP by an assessing doctor.

**Conclusion:** The documentation and communication following a MHAA was not in keeping with GMC GMP guidance and the MHACP in the vast majority of cases assessed. This poses a significant patient safety concern.

